# Time-Lapse Imaging of Neuroblastoma Cells to Determine Cell Fate upon Gene Knockdown

**DOI:** 10.1371/journal.pone.0050988

**Published:** 2012-12-12

**Authors:** Richa Batra, Nathalie Harder, Sina Gogolin, Nicolle Diessl, Zita Soons, Christina Jäger-Schmidt, Christian Lawerenz, Roland Eils, Karl Rohr, Frank Westermann, Rainer König

**Affiliations:** 1 Department of Bioinformatics and Functional Genomics, Institute of Pharmacy and Molecular Biotechnology, Bioquant, University of Heidelberg, Heidelberg, Germany; 2 Division of Theoretical Bioinformatics, German Cancer Research Centre (DKFZ), Heidelberg, Germany; 3 Division of Tumor Genetics, German Cancer Research Centre (DKFZ), Heidelberg, Germany; 4 Department of Genomics and Proteomics Core Facility, High-Throughput Screening, German Cancer Research Center, Heidelberg, Germany; Wayne State University, United States of America

## Abstract

Neuroblastoma is the most common extra-cranial solid tumor of early childhood. Standard therapies are not effective in case of poor prognosis and chemotherapy resistance. To improve drug therapy, it is imperative to discover new targets that play a substantial role in tumorigenesis of neuroblastoma. The mitotic machinery is an attractive target for therapeutic interventions and inhibitors can be developed to target mitotic entry, spindle apparatus, spindle activation checkpoint, and mitotic exit. We present an elaborate analysis pipeline to determine cancer specific therapeutic targets by first performing a focused gene expression analysis to select genes followed by a gene knockdown screening assay of live cells. We interrogated gene expression studies of neuroblastoma tumors and selected 240 genes relevant for tumorigenesis and cell cycle. With these genes we performed time-lapse screening of gene knockdowns in neuroblastoma cells. We classified cellular phenotypes and used the temporal context of the perturbation effect to determine the sequence of events, particularly the mitotic entry preceding cell death. Based upon this phenotype kinetics from the gene knockdown screening, we inferred dynamic gene functions in mitosis and cell proliferation. We identified six genes (*DLGAP5*, *DSCC1*, *SMO*, *SNRPD1*, *SSBP1*, and *UBE2C*) with a vital role in mitosis and these are promising therapeutic targets for neuroblastoma. Images and movies of every time point of all screened genes are available at https://ichip.bioquant.uni-heidelberg.de.

## Introduction

Neuroblastoma is an embryonal tumor arising in the sympathetic nervous system, mostly in adrenal glands. The clinical courses of neuroblastoma are very heterogeneous. Some tumors undergo spontaneous regression without therapy, whereas, high-risk neuroblastoma patients are often resistant to available therapies and undergo a fatal clinical outcome [1]. These different clinical courses depend on age of the patient, stage of the disease and genetic abnormalities, most prominently the amplification of the transcription factor *MYCN* [2]. *MYCN* serves as a prognostic marker for neuroblastoma [3], [4] and is a central regulator of the cell cycle [5]. In addition, mutations in *ALK* [6] and *PHOX2B* [7] have been identified in most familial cases of neuroblastoma. Despite the recent progress in understanding gene function, specific targets for curing neuroblastoma tumors are yet unknown. Deregulation of cell division is a hallmark of cancerous cells [8]. The mitotic spindle is an essential component of cell division that ensures an equal division of the chromosomes [9]. Inhibitors of the mitotic spindle have been extensively used in chemotherapy [9]. However, susceptibility to these drugs is dependent on the tumor type [10]. Though, given the high degree of heterogeneity in response to anti-mitotic drugs in different tumor cells, [11] identification of target proteins that are substantial for the etiology of neuroblastoma is a challenging task.

Hence, the search for genes with therapeutic potential requires an elaborated approach. Neuroblastoma exhibit heterogeneous clinical courses. Stage 4 classified tumors have a very poor prognosis (aggressive tumors), in contrast to stage 1 tumors which have a very good prognosis and often show spontaneous regression [3]. For the present knockdown screen, we selected genes from an established gene-expression-based classifier. This predictor was constructed to discriminate neuroblastoma tumors with poor prognosis (stage 4) from tumors with good prognosis (stage 1, see our earlier study, [12]). Furthermore, we selected genes which are regulated by the prognostic marker *MYCN/MYC* as found in our previous *in vitro* study [4]. In this previous work, a genome-wide search for *MYCN* targets was performed to identify clusters of genes that were directly regulated by *MYC/MYCN* or indirectly involved in *MYCN-*induced regulation, using a neuroblastoma cell line that allows conditional expression of *MYCN*.

Functional genomics and cancer genetics consistently exploit high-throughput RNA interference knockdown screens to investigate consequences of eliminating specific genes [13]–[15]. siRNA assays based on a single readout, such as cell viability, growth rate, or reporter activity (luciferase) are easy to scale up in high throughput. However, they contain limited information as they provide only an endpoint snapshot of a cell's reaction [16]. In contrast, image-based knockdown screens provide multi-parametric readouts and enable tracking more complex phenotypes. These assays are laborious if done on a high-throughput scale. We combined the best of both to infer gene function in a time-dependent manner, as explained in the following. To gain functional information from images, image processing methods were established to segment whole cells and cell nuclei (i.e. to separate them from the image background) and to extract their morphological features [17], [18]. Techniques have been developed to distinguish and quantify different cell shapes [19], to determine sub cellular localizations [20], to identify mitotic phases [21], and to cluster genes based on phenotypic similarity [22].

**Figure 1:**
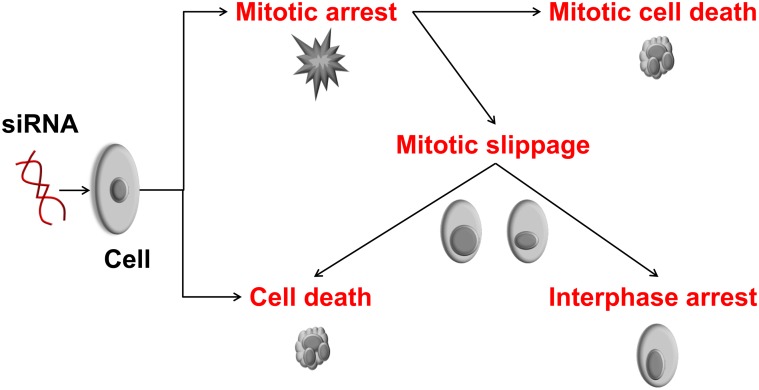
Consequences of a gene knockdown on the cell cycle and cell fate. These effects can be observed (directly or indirectly) by imaging cells with silenced genes following a mitotic time-lapse screening assay. Cells may directly be affected from a loss-of-function of a gene and die (cell death), they may enter mitosis and die before completion of mitosis (cell death in mitotic arrest) or may undergo mitotic slippage followed by interphase arrest or cell death [23].

In this study, we used a reduced set of genes relevant to neuroblastoma, as described above and performed a time-lapse image-based loss-of-function assay to determine cell fate upon gene knockdown. As an example, different outcomes of gene silencing are shown in Figure 1. For instance, perturbation of constitutively expressed anti-apoptotic genes may lead to cell death. As such, targeting mitotic genes can lead to mitotic arrest and this may lead to cell death depending on the mitotic component that was targeted [23], [24]. Targeting the mitotic checkpoint can cause aneuploidy resulting in asymmetric segregation of chromosomes during anaphase. An abnormal division can result in non-viable daughter cells. Some knockdowns can cause mitotic arrest and after prolonged mitotic arrest, a cell can either die or exit mitosis without cell division known as mitotic slippage. Knockdowns resulting in such abnormal mitotic fate are attractive therapeutic candidates. Hence, we focused our analysis on identifying such perturbations.

## Results and Discusson

**Figure 2:**
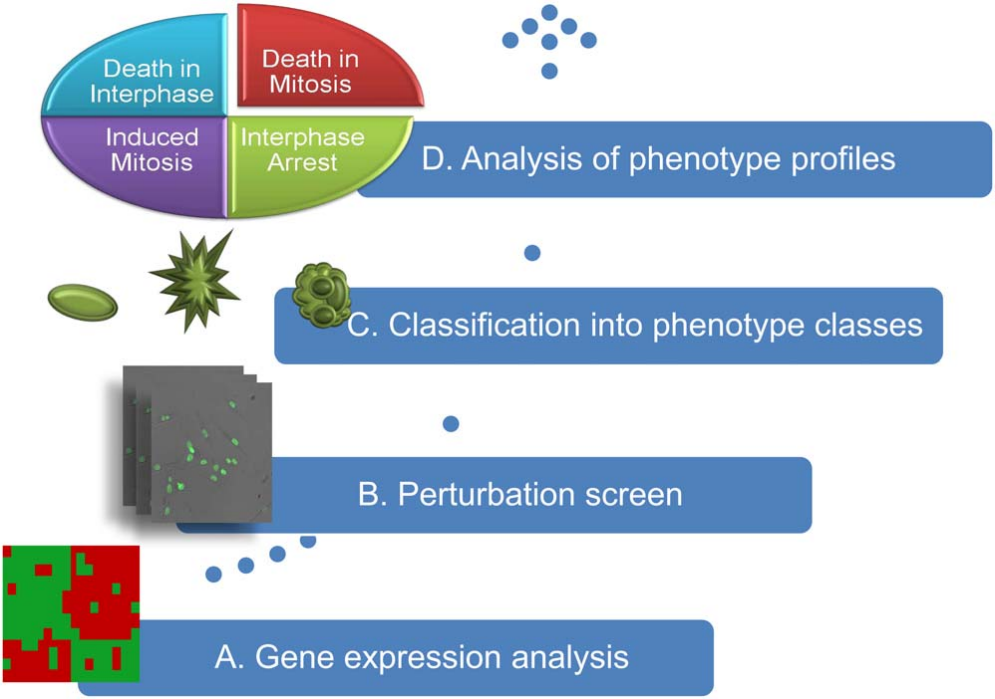
The workflow. (A) Neuroblastoma associated genes were selected based on gene expression profiles of neuroblastoma tumors and cell lines, (B) selected genes were subjected to image-based time-lapse siRNA knockdown screens, (C) each cell in an image was classified into one of the phenotype classes interphase, mitosis, or cell death, and (D) time series of the phenotypes were assembled into phenotype profiles to determine gene function of each gene knockdown.

The workflow is depicted in Figure 2. Firstly, based on gene expression analysis, we selected genes involved in the malignant progression of neuroblastomas. Subsequently, these genes were subjected to time-lapse image-based knockdown screens in the SH-EP cell line from neuroblastoma. By automated image processing and machine learning using Support Vector Machines (SVMs), a quantitative description of phenotypic classes and cell nuclei were obtained from raw bitmaps. Thereafter, perturbation consequence was inferred from the analysis of the phenotypic dynamics focusing on cell death, death in mitosis and death after mitosis. For validation, the analysis was repeated using a second neuroblastoma cell line (SK-N-BE(2)-C). This resulted in a small set of genes which was verified using gene expression data from neuroblastoma patients and literature. Finally, we predicted potential kinases regulating the candidate genes using a repository of kinase-substrate interactions and compared this with the in literature.

### Selecting relevant genes for knockdown screening

In a previous study by Oberthuer *et al.* [12], a predictive-signature comprising of 144 genes was established to predict the course of the disease for neuroblastoma patients. In a follow-up study by Westermann *et al.* [4], a genome-wide search of *MYCN/MYC* target genes using a *MYCN*-inducible neuroblastoma cell line was performed recording time series of gene expression after *MYCN* induction. The profiles were clustered yielding gene sets with similar gene expression profiles. For our screen, we selected two sets of genes from these clusters, one set from clusters enriched in genes that belonged to the 144-gene predictor signature. The second set of genes was selected from clusters enriched (*p-value*≤0.05, adjusted for multiple testing) in the “E-Box” motif (binding motif of MYC family), indicating direct MYC family targets [4]. Details on the selection are given in Methods. The gene list is provided in an addition file (see Supplementary Table S1). Using the selected 240 neuroblastoma associated genes, we performed enrichment tests for the pathway definitions of the Reactome database [25] and Gene Ontology (www.gene.ontology.org). We found four Reactome pathways to be significantly enriched with the candidate genes comprising cell cycle associated pathways (mitotic cell cycle, cell cycle checkpoints and APC-Cdc20 mediated degradation of *Nek2A*) (see Supplementary Table S2). For Gene Ontology, four out of the top five Gene Ontology terms were linked to cell cycle (mitosis, cell division, mitotic spindle organization, and mitotic cell cycle checkpoint), demonstrating that the gene selection procedure captured genes relevant to the cell cycle.

### Automated classification of cellular phenotypes

**Figure 3:**
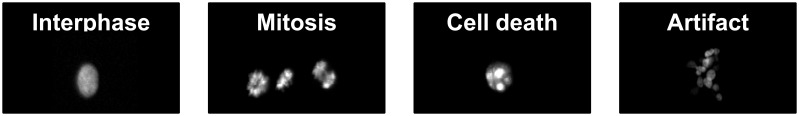
Sample images of the four phenotype classes. Interphase cells are round or elliptical with smooth boundaries. The class of mitosis includes cells in the sub-phases of the mitotic process, i.e. pro-metaphase, metaphase, and anaphase. The class cell death represents dying cells observed by disintegrated nuclei. The class artifact represents cell aggregations that could not be further segmented and over-segmented cells.

Each single cell nucleus was segmented from images and characterized by texture descriptors, e.g. Haralick texture, Zernike moments, granularity, greyscale invariants, Wavelet features and by morphological descriptors, e.g. shape, size and circularity. To track mitotic events, each cell was classified into one out of four distinct phenotype classes (Figure 3), i.e. interphase (round or elliptical object with smooth boundaries), mitosis (dividing cell comprising prometaphase, metaphase, and anaphase), cell death (small and bright fragments of the nuclei), and artifact (clusters of cells that could not be further subdivided, or small-dark objects; these objects were not used for a further functional analysis). The classifier was trained using Support Vector Machines (SVMs) to distinguish these four phenotype classes on a training set of manually annotated nuclei. To assess the performance of the classifiers, a cross-validation procedure was performed, yielding an overall accuracy of 95.3% for the SH-EP cell line. These results outperform previous investigations with HeLa cells (accuracy for HeLa cells: 93.9% to 94.7% [18]) even though imaging and image analysis of neuroblastoma cells was more challenging due to the higher tendency of cells to form cluster and higher cell motility. Note that the stated performance of our approach was determined using well separable objects of the training set.

To determine the performance on all objects, we randomly selected a test set from all segmented objects of our data. Manual verification of the classified phenotypes showed that our classifiers well distinguished the phenotypes (accuracy: 83.7%). Nevertheless, separation of mitosis and interphase, and of mitosis and cell death was comparably low. In order to improve the separation of these challenging cases, we designed an automated correction scheme employing tracking information (see section 2.3). In addition, we applied a filter to discard objects for which the predicted phenotypes were ambiguous. As a result, the filter discarded ∼8% of the objects. These automatic corrections improved overall accuracy from 83.6% to 86.5%. In particular, accuracy for the class mitosis increased considerably: from 81.7% (see Supplementary Table S3) to 93.8% (Table 1). In summary, we obtained reliable results by improving automated classification of phenotypes from image data of neuroblastoma cell lines (results for SK-N-BE(2)-C are given in Supplementary Table S4).

**Table 1:** Confusion matrix of the classification results after automated correction.

			True	class	
		Interphase	Mitosis	Cell Death	Artifact
	Interphase	241	11	10	63
**Predicted**	Mitosis	5	16	12	10
**class**	Cell Death	28	5	119	33
	Artifact	25	0	6	104

### Quality control of the experimental set-up

Knockdown experiments were performed for two neuroblastoma cell lines stably expressing GFP tagged histones using solid-phase reverse transfection with siRNA. Cells were imaged for 120 hours with a time-lapse of approximately 40 min with one image per well. As a quality control, we compared proliferation dynamics of positive and negative controls over the entire period of the screening. We selected positive controls with a distinct apoptotic phenotype as shown elsewhere [26]; in pilot screens, we observed similar phenotypes for three of these genes (*KIF11*, *PLK1*, *INCENP*, data not shown). We used these as positive controls and two scramble siRNA constructs as negative controls. To obtain a measure of proliferation dynamics, we counted the number of interphase cells in each image over the investigated time-frames. For the SH-EP cell line, we found a significantly reduced proliferation of the positive controls in comparison to the negative controls in all time-frames (*p-value*≤0.05, see Supplementary Figure S1). For the other cell line (SK-N-BE(2)-C), we found a significantly reduced proliferation in the later time-frames (56–120 hours, see Supplementary Figure S1) indicating a delayed effect of the perturbation.

### Estimating cell cycle kinetics

**Figure 4:**
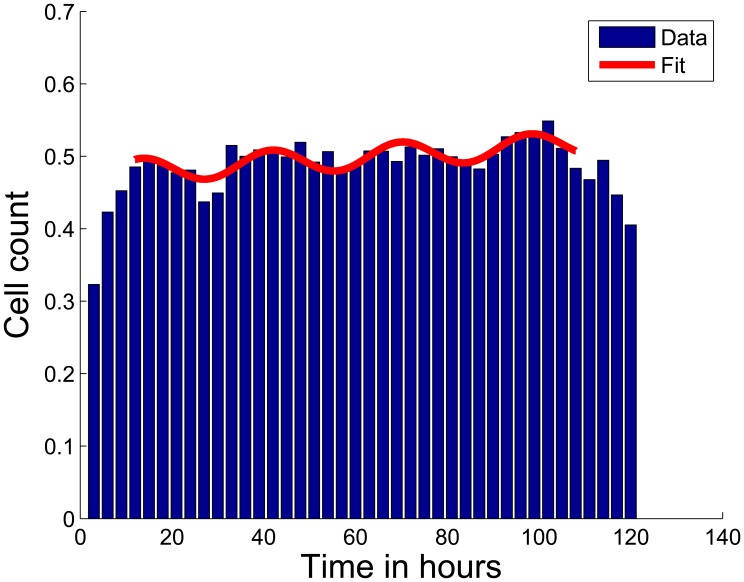
Time series of interphase cells during five days of screening. The population shows a periodicity of ∼35 hours representing the cell cycle duration (blue bars: interphase counts (normalized by B-Score normalization) of all screened cells for each time-frame, red curve: fitting curve).

Cell cycle kinetics has been used as a parameter for optimization of cancer treatment schedules. Interestingly, treatment schedules matching the integer multiple of the cell cycle duration reduce damage to normal cells [27]. Hence, we were interested if our time series analysis allowed us to estimate the cell cycle duration of our cell lines. We examined the cell cycle behavior of the cell culture, assuming that siRNA transfection causes synchronization of the cells. The cell cycle duration of a cell line can be computed either by the mitotic index or by S-phase dynamics [28]. In our approach, interphase phases G1, G2, and S were not differentiated therefore we studied the interphase dynamics as a whole. The interphase population was averaged over all replicates and knockdowns. In accordance with our expectation we observed periodicity. We identified a cell cycle duration of ∼35 hours for SH-EP cells (Figure 4) and of ∼31 hours for SK-N-BE(2)-C (Supplementary Figure S2). Note that in earlier studies using HeLa cells, a shorter cell cycle duration of 17 hours was reported [29]. Our finding shows that neuroblastoma cells synchronize as well as it opens the possibility to study population response dynamics for each knockdown (next sections).

### Identifying knockdowns that impair cell cycle

**Figure 5:**
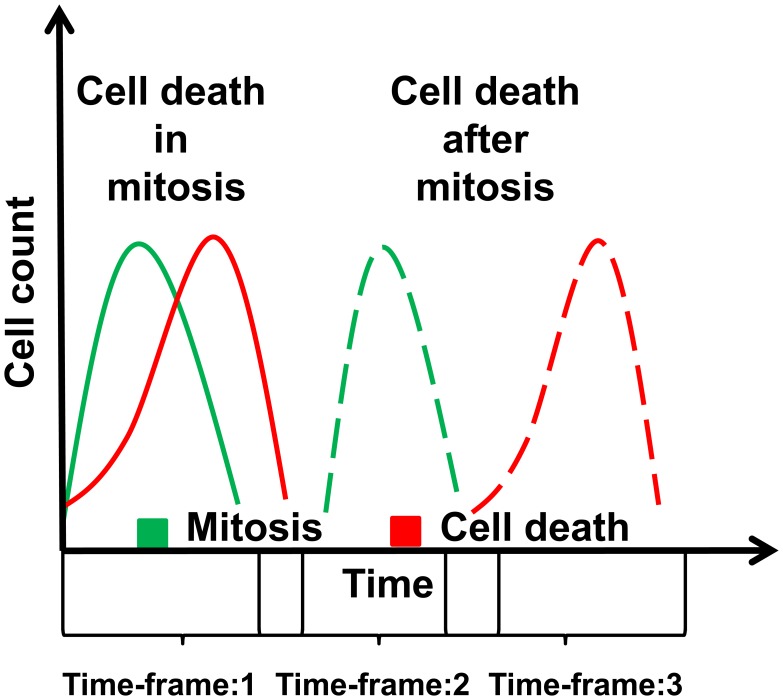
Determination of candidate genes that show cell death during or after mitosis. The population response to a knockdown was computed to identify the sequence of phenotype occurrence.

Death in mitosis, i.e. cell death before completion of the mitotic process, has been reported as the most promising component in cell cycle for drug design [23]. This can be explained by the concept that inhibitors affecting the initial phase of the cell cycle lead to cells in quiescence. Inhibitors leading to high cell death in general also affect normal cells causing severe side effects during therapy. Therefore, we tracked the sequence of phenotypes in the population to select genes either with a high number of cells in mitosis and cell death at the same time-frame or a high number of cells in mitosis followed by cell death (Figure 5).Note that the phenotype was observed in the context of the population response in a time-frame. Accordingly, these genes either showed mitotic cell death or mitotic slippage preceding cell death.

### Comparing the two neuroblastoma cell lines

We found 30 candidate genes as potential drug targets (using the statistical analysis pipeline for monitoring phenotype dynamics as described in Methods) of the SH-EP cell line (see Supplementary Table S5). As a validation, we compared the results with the second cell line which was subjected to the same screening protocol (SK-N-BE(2)-C, results in Supplementary Table S5). Six identified genes (*DSCC1*, *DLGAP5*, *UBE2C*, *SSBP1*, *SNRPD1*, and *SMO*) were validated by the second cell line. The overlap showed a potential enrichment (*p-value* = 0.14). We did not find a corresponding phenotype in a genome-wide HeLa cell screen (Mitocheck database, http://www.mitocheck.org/cgi-bin/mtc).

### Gene expression analysis of the validated genes

Interestingly, all these genes were highly up-regulated (*p-value*<0.01, see Supplementary Table S6) in aggressive neuroblastoma tumors (stage 4, with *MYCN* amplification) in comparison to non-aggressive tumors (stage 1 without *MYCN* amplification). Furthermore, all six genes showed a good prediction performance for overall survival (see Supplementary Table S6). Kaplan Meier plots for *SMO* and *DLGAP5* are shown in Supplementary Figure S3.

### Literature reports of the validated genes

A functional interpretation of the six identified genes is given the following: (1) *DLGAP5* (Discs, Large homolog-Associated Protein 5) is a known mitotic regulator. It stabilizes microtubules and ensures bipolar spindle formation. *AURKA* regulates its activity by phosphorylation [30]. *DLGAP5* depleted HeLa cells have shown a delay in mitotic progression and their mitotic exit resulted in an unequal segregation of chromosomes [31]. (2) *DSCC1* (Defective in Sister Chromatid Cohesion 1 homolog) is one of the components of the replication factor C (RFC) complex with an important role during S phase of the cell cycle [32]. *DSCC1* double mutants terminated proliferation and showed premature senescence (increased size, flattened morphology) [33]. (3) *SMO* (Smoothened) is a G protein-coupled receptor that interacts with *PTCH*, a receptor for hedgehog proteins. The hedgehog signaling pathway regulates cell proliferation, differentiation and tissue patterning during embryonic development [34]. *SMO* has been identified as a potential drug target in osteosarcoma, as its inhibitor cyclopamine promotes G1 arrests and represses expression of *cyclin D1*, *cyclin E1*, *SKP2*, and *pRb* [35]. Deregulation of the hedgehog signaling pathway has been discovered in brain, lung and skin cancers [34]. Inhibitors targeting *SMO* for curing medulloblastoma tumors are in clinical trials [36]. (4) *SNRPD1* encodes a small nuclear ribonucleoprotein that belongs to the SNRNP core protein family. It acts as a charged protein scaffold to promote SNRNP assembly and it strengthens SNRNP-SNRNP interactions through non-specific electrostatic contacts with RNA [37]. snRNPs are major components of the spliceosome [38]. (5) *SSBP1* (Single-Stranded DNA Binding Protein 1) is a housekeeping gene associated with mitochondrial biogenesis. It interacts with tumor-suppressor *TP53* to enable DNA repair in mitochondria during oxidative stress [39]. Its inhibition causes genomic instability and negatively affects cell cycle checkpoint activation [40]. (6) *UBE2C* is an E2 ubiquitin-conjugating enzyme. It is required for degradation of mitotic cyclins and for cell cycle progression [37]. Its knockdown in U251 glioma cells results in arrest at G2/M phase and apoptosis through induction of Bax and p53 [41]. Supplementary Figure S4 depicts a selection of typical time-lapses of cells with these gene knockdowns. Note that the cell responses were heterogeneous for all investigated gene knockdowns and thus we based our analysis on population response. In summary, four (*DLGAP5*, *DSCC1*, *SSBP1*, *UBE2C*) of these six proteins are directly involved in cell cycle and one indirectly (*SMO*) which is involved in cell cycle regulation. As such, the functional interpretation of the six candidate genes provides strong indications that monitoring cell cycle dynamics enables identification of drug targets for neuroblastoma cells.

### Predicting upstream regulators

Protein phosphorylation by kinases is a common regulatory mechanism in signaling of cell cycle progression and mitotic processes. The fact that most tumors show alterations herein makes kinases attractive therapeutic targets [42]. We performed statistical enrichment analysis (using KEA [43]) for the proteins encoded by the genes with mitosis-linked cell death phenotype, as potential substrates of regulatory kinases (see Supplementary Table S7). In both cell lines, the Aurora kinase family showed a significant enrichment of substrates among our candidate genes. For the SH-EP cell line, the top three kinase families identified were AUR, GSK and CDK (*p-value:* 0.0003, 0.005 and 0.006, respectively). Interestingly, we found the GSK family, which has not been associated with neuroblastoma therapy as prominently as the CDKs and AURs. The family of GSKs consists of multifunctional serine-threonine kinases *GSK3α* and *GSK3β* [44]. Their role in cancer and chromosome assembly on the metaphase plate has been recently discovered [45]–[47]. It has been shown that *GSK3β* inhibition leads to G2/M accumulation and increased apoptosis in the neuroblastoma cell line SK-N-SH [48]. In glioma cells, inhibition of GSK3 induces pro-apoptotic effects, inhibits pro-survival signals, and induces mitochondrial permeability [49]. GSK target genes among our candidate genes are *NIFK*, *LMNB1*, *NCL*, *SMARCC1* and *TP53*. Detailed functional interpretation of the other kinases we identified and the downstream targets of GSKs are given in Supplementary Text S1.

### Data access via data repository iCHIP

All original data from this study is publicly available in the web-based database iCHIP. It can be accessed at https://ichip.bioquant.uni-heidelberg.de (User: “guest”; Password: “sHeY82Nu”). Each movie and each image can be observed and downloaded. Access to the images is achieved by selection of a gene in the query page. Associated gene, siRNA information as well as the calculated phenotype scoring and related quality measures are available. Supplementary Figure S5 shows a screenshot of an exemplary webpage at which a movie can be viewed and downloaded.

## Conclusion

We have developed a processing pipeline for screens by time-lapse microscopy from raw bitmaps to detailed perturbation analysis and identification of drug targets for tumors. As a case study, we applied the pipeline to neuroblastoma cells. The methodological contributions are threefold. First, integration of gene expression and gene knockdown analysis enables overcoming challenges posed by large genome-wide time-lapse studies. Second, optimization of the classification of cellular phenotypes enhances their correct prediction. Third, a novel analysis technique to track knockdown phenotype kinetics makes it possible to monitor cellular decisions during cell cycle.

Genome-wide siRNA screens are costly, need large data storage capacities, are very time consuming, and may still lead to ambiguous results (amongst others, see [50]–[53]). In contrast, kinome screens are less resource intensive and focus on a subset of genes (kinases) of the human genome [14], [54]. In line with kinome screens, we focused our screen on a set of genes which are involved in cell cycle progression and tumorigenesis of neuroblastoma cells. We identified a list of candidates by analyzing large sets of publicly available gene expression data. From this list, we selected a set of 240 genes for knockdown studies, which have a potential role in neuroblastoma tumor progression.

To track mitotic aberrations after gene knockdown, we monitored well defined phenotypic classes (interphase, mitosis, cell death) of cell nuclei. In the proposed pipeline, classification of the cells into distinct phenotypes using image-based screens was crucial, as any follow-up interpretation based upon this. We did not solely rely on the cross-validation accuracy values to assess classification performance. Instead, the classified phenotypes were manually evaluated on a randomly selected test set. In addition, assignment of the mitosis and interphase classes was improved by an automated correction scheme employing tracking information. Subsequently, these phenotypes were analyzed in a time dependent manner. Tracking mitosis with a time-lapse of ∼40 min at a single cell level was a challenging task. Our finding that neuroblastoma cells synchronize their cell cycle opens the possibility to monitor phenotype kinetics using population response. We tracked population response and observed the consequence of gene perturbation considering integration of overlapping time-frames. In the end, we identified six genes (DLGAP5, DSCC1, SSBP1, UBE2C, SNRPD1, and SMO) with a vital role in prevention of cell death in both cell lines and hence six potential drug targets for silencing in cancer therapy. These genes were significantly up-regulated in aggressive neuroblastoma tumors and are good predictors for clinical outcome. In this study, we employed the neuroblastoma cell lines SH-EP and SK-N-BE(2)-C. As a future aspect, our findings need validations using a larger set of different neuroblastoma cell lines and cells from primary tumors. In summary, we developed a general method to characterize cell fate upon knockdown using high-throughput time-lapse image data, and applied the pipeline to neuroblastoma cells. The analysis identified six novel candidates which were not previously associated with cell cycle in neuroblastoma cells. With a detailed analysis of the phenotypic dynamics, we hope to elucidate the central players for the cellular decision during tumorigenesis in neuroblastoma.

## Methods

### Selecting genes for screening using gene expression analysis

In a previous study by Oberthuer *et al.* [12], a neuroblastoma-specific microarray chip was designed which covered a high percentage of transcripts that were differentially expressed in the major clinically distinct subgroups of neuroblastoma tumors. Using this customized 11K oligonucleotide microarray, 251 neuroblastoma specimens were analyzed and a 144-gene predictor signature was assembled to predict the course of the disease. In a follow-up study by Westermann *et al.* [4], the same neuroblastoma-specific microarray was used to identify *MYCN*/*MYC* target genes using a neuroblastoma cell line (SH-EP*^MYCN^*). SH-EP*^MYCN^* is a neuroblastoma cell line that stably expresses an inducible *MYCN* transgene, thus allowing conditional expression of *MYCN*. Gene expression profiles of a time series after *MYCN* induction were obtained with the customized 11k microarray. The profiles were clustered using self-organizing maps (SOM) which resulted in 504 clusters (best matching units, BMUs) of genes with similar gene expression profiles. Clusters (BMU 140, 168, 195, 280, 308, 336, defined as subgroups I and II in [4]) were enriched in the “E-Box” motif (binding motif of *MYCN/MYC*, *P*≤0.05 using a Fisher's Exact test, adjusted for multiple testing of all BMUs using the method of Benjamini-Hochberg [55]), indicating potential targets of the MYC transcription factor family [4]. We selected 127 genes from these clusters. In addition, we selected 80 genes from the BMUs which were enriched in genes from the 144-gene predictor signature. For this, we computed the percentages of the predictive-signature-genes that matched to the identified clusters. The top three BMUs (BMU 504, 476, 475) with the highest odd ratios (0.49, 0.41, and 0.3) were selected. Further, 33 genes which were associated to neuroblastoma tumor progression were selected from literature. Finally a set of 240 genes was assembled and used for the knockdown screen (see Supplementary Table S1).

### Preparation of cell arrays and imaging

Two neuroblastoma cell lines, SH-EP and SK-N-BE(2)-C, were used in the screen. These cell lines were transfected with a construct of the gene coding for histone H2B with Green Fluorescent Protein (GFP) as described previously [56]. Briefly, a chimeric gene with a cDNA construct of H2B gene tagged with GFP was sub-cloned into a mammalian expression vector. This vector was used to transfect the cell lines. Thus, the product of this gene H2B-GFP protein was incorporated into the nucleosomes which allowed imaging of mitotic chromosomes and interphase chromosomes. Further, cover glass culture chambers called “LabTeks” were automatically spotted and dried as previously described [57]. Sample preparation for spotting, mixing of transfection reagents and siRNAs was done using an automated liquid handler. Automated spotting of this transfection solution onto LabTeks was performed with a contact printer. After drying the LabTeks for at least 12 hours, SH-EP/H2B-GFP and SK-N-BE(2)-C/H2B-GFP (60, 000 cells/LabTek) were seeded on the LabTeks and incubated in a stage top chamber by *LCI*, with 1.5 ml growth medium at 37°C, 95% humidity, and 5% CO_2_. Eight LabTeks with 275 spots were used to cover several mock (no siRNA) spots, 2 siRNAs (Ambion) per gene and four replicates per siRNA. Images were acquired (16 hour post seeding) for five days at an acquisition rate of 35–40 minutes using an automated wide-field fluorescence microscope (*Olympus X81 ‘inverted’ ScanR System*) with 10× magnification.

### Image Processing

Nuclei segmentation was performed using a region adaptive threshold scheme, which allowed detection of cells with varying contrast. Clusters of cells were resolved by Euclidean distance transformation of the segmentation output followed by watershed transformation to split them into single cells. Dense clusters of cells growing on top of each other could not be resolved using this approach and were treated as cluster objects in the subsequent analysis. To bring all images of different spots and cell arrays to a comparable grey value range, grey value normalization was performed before feature extraction. To this end, the mean of the distribution (histogram) of the foreground pixels of the complete data set was computed and three features of this histogram were extracted (i.e. location of the maximum peak and its width to the left as well as to the right). For grey value normalization each individual image histogram was mapped to this mean histogram and the grey values of the respective images were transformed and scaled accordingly. A set of 349 image features was computed for each nucleus, describing the texture (Haralick texture, granularity, greyscale invariants, wavelet features) and morphology (shape, size, circularity, Zernike moments), as described previously [17]. Single cell tracking was done based on the approach described in [17]. In essence, first cell-cell correspondences were determined using spatial distance and feature similarity, and second, mitosis events (cell splitting) were detected and the respective trajectories were merged [17], [18]. To determine cell-cell associations a distance measure was used, combining feature similarity and spatial distance after normalization of both terms [17]. For mitosis detection the mitosis likelihood function as described in [18] was used, which is based on the sizes and mean intensities of the mother and daughter cell nuclei. An additional constraint was added to the mitosis likelihood function, disregarding objects with low mean intensity (compared to the mean object intensity in the particular image) to avoid false positive detections.

### Classification of nuclei into phenotypes

Using supervised machine learning, each nucleus was classified into one of the following phenotype classes: interphase, mitosis, cell death, and artifact (Figure 3). For training of the classifier a set of typical training samples from each class was collected, where each sample was defined by a vector of descriptive image features (e.g., Haralick texture, Zernike moments, Wavelet features, shape descriptors) and a class label (interphase, mitosis, cell death, artifact). The class label was provided by the annotation of an expert. A classification model (classifier) was generated from the training data to distinguish the classes defined in the training set. After training, the classifier was applied to assign class labels to nucleus images for which the classes were not yet known. Each step in this process is explained in the following.

### Training set

The training set was manually annotated by an expert. For the SH-EP cell line, a set of 174 interphase samples, 94 mitosis samples, 204 cell death samples, and 118 artifact samples, was manually annotated for training and validating the classifier. For SK-N-BE(2)-C cells, we selected a set of 230 interphase samples, 80 mitosis samples, 120 cell death samples, 100 cluster samples, and 45 artifact samples. Since SK-N-BE(2)-C showed a much higher tendency of clustering, we separated the clustered objects from the artifact class and defined a new class called cluster. These samples were taken from the images of all of the eight LabTeks to account for the variation among the cell arrays of the entire screen. The imbalanced training set was stratified for the classifier by weighting each sample of class *c* by *w_c_* = *n_l_/n_c_* as described in [18]. *n_l_* is the number of samples in the largest class and *n*_c_ is the number of samples in class *c*.

### Classification model

For classification we used Support Vector Machines (SVMs) with a radial basis function (RBF) kernel. We applied a one-against-one approach for multiclass classification (i.e. binary classification between all pairs, followed by voting) as implemented in the R-package e1071 [58]. The model parameters *C* (cost function) and γ (kernel width) were optimized by a grid search *C* = {2^1^, 2^2^,….2^10^}, γ = {2^−16^,2^−15^…2^−6^} employing a 10-fold cross-validation on the training data (inner loop). To choose C and γ, each pair of the parameters C and γ was tested. The pair with the lowest validation error (the average number of misclassified samples) was chosen and used for training an SVM on the complete training dataset. To estimate the performance of the classifiers, the SVMs were trained and validated by a 5-fold cross-validation (outer loop). The annotated data was split into five subsets, four subsets were selected as training data and the remaining subset as test data. The whole process was repeated 5 times (outer loop) yielding performance estimations of the classifiers. For classifying new samples, new SVMs were trained with all samples from the training data.

### Filter

Cells which could not be assigned to any phenotype with high confidence were removed based on the likelihood for their respective class label as determined by the classifier. The confidence values were obtained using the R-package e1071. A probability model was used which computes *a posteriori* probabilities for the multi-class problem by a quadratic optimization [59]. This provides the likelihood of each class label for a sample. For ambiguous samples the likelihood values for multiple classes were similar without a clear maximum, and consequently, the classifier output was less reliable. Therefore, we defined a reliability score *r*; which was computed for each sample by *r* = |*l_1_−l_2_*|, where *l_1_* and *l_2_* are the two highest likelihood values (predicted by the SVMs). All samples with a reliability score of *r*≤0.2 were discarded from the further analysis.

### Manual evaluation of classification

For evaluating the performance of the classifier on real data (including samples which were hard to distinguish), a set *s* of 800 nuclei was randomly selected which included samples from each class. Set *s* was classified using the above model and filter. Independently, this set was manually annotated. Single cell tracking as described in [17] was used to extract the trajectory *tr* of each of the selected nuclei of *s*. *tr* of a nucleus consisted of three snapshots before and three after the target snapshot (i.e. the snapshot which is a part of *s*) and this time series was used for supporting the manual annotation of the nuclei into phenotype classes. The two labels of the samples (manual annotation, classifier) were compared. These errors were studied to formulate the correction rules as described below.

### Automatic error correction

Classification correction was performed based on a finite state model (FSM) as described previously [18] which is described briefly in the following. Each cell was tracked over the whole time series as previously explained [17], [18]. Classification results were overlaid on these trajectories resulting in a sequence comprising phenotype classes of a nucleus over time. A correction scheme was developed for better separating the class mitosis from interphase and cell death. This automatic correction scheme was aimed at (1) avoiding false negative prediction of mitosis, (2) avoiding false positive prediction of mitosis, and (3) avoiding false positive prediction of cell death. For (1), all splitting events were identified, and then the mother nucleus as well as the immediate daughter nuclei were labeled mitosis. For (2), all nuclei classified by the classifier as mitosis were validated by inspecting any of the four conditions: (a) if it was involved in a splitting event (mother or daughter), (b) if there was a splitting event preceding or following the nucleus, (c) if the succeeding object was a cluster (a mitotic splitting event would not be detectable in a cluster), or (d) if it was followed by cell death. If none of the conditions were true, the nucleus was corrected to interphase. For (3), all the successors of the nucleus were scanned until the end of the trajectory. A nucleus was considered to be in cell death if the immediate successor of the nucleus and at least 50% of the following trajectory had the label cell death, if not, the sample was corrected to interphase.

### Quantitative analysis of phenotype kinetics

After classifying each nucleus, we performed a quantitative analysis to obtain time-lapse profiles for each phenotype class and knockdown. The pipeline included the following steps:

### Normalization

We used B-Score normalization for normalization within the LabTeks and between LabTeks, accounting for spatial error corrections of each cell array per time-lapse and per phenotype class. B-Score normalization subtracts the row mean and column mean to account for the row and column variability, followed by correction for plate deviations by subtracting the plate mean and dividing by the plate median absolute deviation [60], i.e.
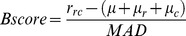
(1)where *Bscore* is the normalized value, *r_RC_* is the original value of the plate at row *R* and column *C*, *μ* is the mean of the plate, *μ_R_* is the mean of row *R*, *μ_C_* is the mean of column *C*, *MAD* is the median absolute deviation of the plate.
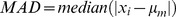
(2)where *x_i_* is the vector of values, *μ_m_* is the median of *x_i_*. Note that, the median absolute deviation is more robust than the standard deviation as the median is less sensitive to outliers [61]. B-score normalization also accounted for edge effects which were evident in the cell arrays before normalization (see Supplementary Figure S6).

### Defining the phenotype signal

To smooth fluctuations, each phenotype class was quantified in time-frames with 24 hours of imaging data. Each time-frame had a shift of 8 hours from the previous frame, yielding 13 time-frames for the five days of screening. The area under the curve (AUC) (integral of the phenotype counts for each time-frame) was computed for each of these time-frames. AUC of a time-frame was defined as the phenotypic signal for that time-frame. AUCs were computed using R-package caTools [62].

### Estimating the phenotypic score

We assigned a significance score to the phenotype signal of each time-frame in the form of *p-values*. We computed significance values (*p-values*) by a non-parametric test (Wilcoxon rank test) instead of using Z-scores, as a significant *p-value* (≤0.05) indicates reproducibility of the siRNA effect and are less sensitive to outliers [63]. The two populations subjected to the test were four replicates of a gene per siRNA, and the overall population acting as the negative control.

### Tracking of the phenotype profiles

Genes which showed a high mitosis count as well as a high count of cell deaths were further investigated to determine the sequence of the occurrence of these phenotypes. A phenotype profile of a gene consisted of the *p-value* (Wilcoxon rank test, *p-value*≤0.05) of each time-frame for the two phenotypes under consideration. Mitotic defects were indicated by the occurrence of phenotypes in the same time-frame or occurrence of high cell death in the time-frame next to the time-frame with high mitosis counts (Figure 5). We selected the genes with significantly high occurrence of mitosis phenotypes in time-frame t_0_ and significantly high occurrence of cell death phenotypes either at the same time-frame (t_0_) or at time-frame t_0+1_.

### Estimating the periodicity

To estimate the periodicity of the cell lines, we performed a non-linear fit to the overall temporal distribution of all interphase counts (including all controls and knockdowns), using the nlinfit function of Matlab (www.matlab.com). To smooth the data, the entire time series was reduced to forty time-frames. Each frame represented integration of three hours of imaging data. For the fit, a non-linear function combining a sinus function and a linear function was used,
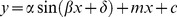
(3)where *α* is the amplitude, *β* the time period, *x* the interphase counts in a time series, δ the phase, *m* the linear slope and *c* a constant. Fitting values of the parameters were *α = 0.03*, *β = 0.18*, *c = 0.4*, *m = 0.001* for SH-EP cells and *α = 0.02*, *β = 0.2*, *c = 0.3*, *m = 0.05* for SK-N-BE(2)-C cells.

### Expression analysis of the validated candidate genes

Gene expression profiles for all the validated genes were extracted from whole genome single-color microarray profiles of 478 pre-treatment primary neuroblastoma tumors analyzed as part of the MAQC-II project [64]. Data was normalized using the quantile method using the R-package limma [65]. Two tumors were removed from the survival analysis as the overall survival data and cause of death were unknown. To split the tumors into high and low risk groups we used the R-package maxstat [66]. We used a 10 fold cross-validation, i.e. we divided the data set into 10 parts and used the cutoff value from 9 parts to assign the group label to the tumors of the 10^th^ part. Overall survival analysis was performed using the R-package survival [67]. Statistical significance of the curves was determined using the log-rank test.

### Enrichment Analysis

Enrichment tests were done for each pathway in Reactome on the screened genes compared with all genes from the 11k microarray as background (universe) using the software DAVID [68]. EASE Scores (from a modified Fisher's exact test) were used for obtaining the significance values [69]. Gene Ontology enrichment analysis was performed using the Bioconductor package topGO [70] and the weight algorithm. Kinase enrichment analysis was done using the Kinase Enrichment Analysis (KEA). It employs a kinase-substrate database, compiled from several experimental resources (for details, see [43]). Given a list of genes, KEA identifies kinases for which a significant enrichment of their substrates can be found in the gene list (using Fisher's exact tests). *P-values* from all these enrichment tests were corrected for multiple testing using the method of Benjamini-Hochberg [55]. After multiple testing corrections, *p-values*≤0.05 were considered to be significant.

## Supporting Information

Text S1 **Predicting upstream regulators.**
(DOCX)

Figure S1 **Experimental quality control.** Top: cell counts of positive (coral red) and negative (coral blue) controls are plotted by boxplots for all time-frames. Bottom: Significance values of the differences of positive and negative controls are given for each time-frame by negative log_10_ *p-values*. A significance threshold (*p-value* = 0.05) is indicated by a red dashed line. (Left) SH-EP cell line: The positive controls show significant lower counts for all time-frames. (Right) SK-N-BE(2)-C cell line: The positive controls show significant lower counts for time frames ≥56 hrs.
(PDF)

Figure S2 **Time series of interphase cells during five days of screening of SK-N-BE(2)-C.** The population showed a periodicity of ∼31 hours representing its cell cycle duration (blue bars: interphase counts of all screened cells for each time frame, red: fitting curve).
(PDF)

Figure S3 **Kaplan Meier plots for two of the validated candidate genes (SMO and DLGAP5).** The log-rank *p-values* are shown on the top right of the plots.
(PDF)

Figure S4 **Selection of time-lapse images illustrating cell fate observed in the SH-EP cell line for the six validated genes.** The image sequence of knockdown of DSCC1 shows a cell in interphase, mitosis (metaphase), interphase (daughter nuclei), deformation of the nucleus (cell death), and cell death. The sequence of knockdown of SSBP1 shows a cell in interphase, mitosis (prometaphase), mitosis (metaphase), mitosis (anaphase), and finally daughter nuclei sticking together in arrest. The sequence of knockdown of SNRPD1 shows a cell in interphase, mitosis (prometaphase), mitosis (metaphase), daughter nuclei and cell death. The sequence of knockdown of UBE2C shows a cell in interphase, mitosis (prometaphase), mitosis (anaphase), and cell death. The sequence of knockdown of DLGAP5 shows a cell in interphase, mitosis (metaphase), daughter nuclei, deformation, and cell death. The sequence of SMO knockdown shows a cell in interphase, mitosis (prometaphase), mitosis (metaphase) and cell death.
(PDF)

Figure S5 **Screenshot of the web interface of the ichip database.** Each movie and each image can be observed and downloaded. Access to the images is achieved by selection of a gene in the query page. Associated gene and siRNA information is also available as well as the calculated phenotpye scoring and related quality measures.
(TIFF)

Figure S6 **Cell arrays before and after normalization.** The color key shows the distribution of the cell counts over the array. Left: A cell array before normalization, showing the edge effects with high cell counts in the most upper row. Right: The same cell array after B-score normalization. It shows a smoothing of the edge effects. Blue boxes represent empty spots which were not a part of the screen.
(PDF)

Table S1 **240 genes selected for knockdown screen.**
(XSLX)

Table S2 **Pathways of Reactome and gene groups from Gene Ontology which were enriched in the screened genes.**
(DOCX)

Table S3 **Confusion matrix for SH-EP cell line.**
(DOCX)

Table S4 **Confusion matrix for SK-N-BE(2)-C cell line.**
(DOCX)

Table S5 **Candidate genes with phenotype Cell death during or after mitosis.**
(DOCX)

Table S6 **Results of the gene expression analysis for the six identified genes.**
(DOCX)

Table S7 **Kinase families and their predicted substrates from our candidate genes.**
(DOCX)
