# Supplementary material for: Time-Lapse Imaging of Neuroblastoma Cells to Determine Cell Fate upon Gene Knockdown
Source: PLoS One. 2012 Dec 12;7(12):e50988. doi: 10.1371/journal.pone.0050988 (PMC3521006; doi:10.1371/journal.pone.0050988)
Supplement: Text S1 — Predicting upstream regulators. (DOCX) [file pone.0050988.s001.docx]

Supplementary Text S1: **Predicting upstream regulators**

Here, we discuss the results of the SH-EP cell line. The top three kinase families identified were AUR, GSK and CDK (*p-value:* 0.0003, 0.005 and 0.006, respectively). The aurora kinase family includes Aurora kinases A, B and C. These are known to regulate cellular division, chromosome segregation, spindle integrity and centrosome regulation [[1](#_ENREF_1),[2](#_ENREF_2)]. Aurora kinase inhibitors have a strong therapeutic potential. Inhibitors of these kinases have been designed and clinical trials are undergoing [[3](#_ENREF_3)].MLN8054 is a selective inhibitor of *AURKA* which is in preclinical trials for neuroblastoma. *AURKB* can be inhibited by AZD1152 which is selectively cytotoxic to neuroblastoma tumor-initiating cells. This inhibitor currently is in a clinical trial for acute myelogenous leukemia [[4](#_ENREF_4)]. Cyclin-dependent kinases (CDKs) require cyclin subunits for their kinase activity. CDKs include *CDK* 1-14, 16, 20 and they are involved in each phase of the cell cycle. CDKs are often deregulated in cancer and have been extensively studied as therapeutic targets. Their inhibitors are in clinical trials [[5](#_ENREF_5),[6](#_ENREF_6),[7](#_ENREF_7)]. Roscovitine is a selective CDK inhibitor. It is in late phase-II trials against non–small cell lung cancer and nasopharyngeal cancer. Roscovitine and its analog CR8 induce cell death in neuroblastoma cells by down-regulating the CDK dependent survival factor Mcl-1[[8](#_ENREF_8)]. Interestingly, we also found the GSK family, which has not been associated with neuroblastoma therapy, as prominently as the CDKs and AURs. The family of GSKs consists of multifunctional serine-threonine kinases *GSK3α* and *GSK3β [*[*9*](#_ENREF_9)*]*. Their role in cancer and chromosome assembly on the metaphase plate has been recently discovered [[10](#_ENREF_10),[11](#_ENREF_11),[12](#_ENREF_12)]. It has been shown that *GSK3β* inhibition leads to G2/M accumulation and increased apoptosis in the neuroblastoma cell line SK-N-SH[[13](#_ENREF_13)]. In glioma cells, inhibition of GSK3 induces pro-apoptotic effects, inhibits pro-survival signals, and induces mitochondrial permeability [[14](#_ENREF_14)]. GSK target genes among our candidate genes are *NIFK*, *LMNB1*, *NCL*, *SMARCC1* and *TP53*. ***NIFK*** interacts with the forkhead-associated domain of the Ki-67 antigen in a mitosis-specific manner. It is a putative RNA-binding protein and may play a role in mitosis and cell cycle progression [[15](#_ENREF_15)]. ***LMNB1*** belongs to the lamin protein family that forms the nuclear membrane. Lamins are essential for various nuclear functions, like the assembly of the nuclear envelope, and DNA replication, and they provide structural integrity and support[[16](#_ENREF_16)]. Depletion of lamin B results in a disorganized spindle and spindle poles, chromosome missegregation and prolonged prometaphase [[17](#_ENREF_17)]. ***NCL*** (nucleolin) is a phosphoprotein abundantly found in the nucleolus. It is involved in ribosome biogenesis, cell proliferation and growth, embryogenesis, cytokinesis and nucleogenesis [[18](#_ENREF_18),[19](#_ENREF_19),[20](#_ENREF_20)]. ***SMARCC1*** is a member of the SWI/SNF family of proteins, with helicase and ATPase activities. Its transitional inactivation and reactivation is required for the formation of a repressed chromatin structure during mitosis [[21](#_ENREF_21)]. It has been associated with colorectal cancer and outcome of the disease. High levels of *SMARCC1* proteins are associated with better overall survival [[22](#_ENREF_22)]. ***TP53*** is well known to regulate cell cycle arrest, apoptosis, senescence, DNA repair, and changes in tumor metabolism [[23](#_ENREF_23)]. More details on the functional interpretation of these genes are given in. In summary, the three families of kinases are substantially involved in regulation of the cell cycle and have therapeutic potential. The substrates of these kinases need to be explored further for their role in neuroblastoma and its therapy.

and cell death. The sequence of knockdown of SSBP1 shows a cell in interphase, mitosis (prometaphase), mitosis (metaphase), mitosis (anaphase), and finally daughter nuclei sticking together in arrest. The sequence of knockdown of SNRPD1 shows a cell in interphase, mitosis (prometaphase), mitosis (metaphase), daughter nuclei and cell death. The sequence of knockdown of UBE2C shows a cell in interphase, mitosis (prometaphase), mitosis (anaphase), and cell death. The sequence of knockdown of DLGAP5 shows a cell in interphase, mitosis (metaphase), daughter nuclei, deformation, and cell death. The sequence of SMO knockdown shows a cell in interphase, mitosis (prometaphase), mitosis (metaphase) and cell death.

References

1. Carmena M, Earnshaw WC (2003) The cellular geography of aurora kinases. Nat Rev Mol Cell Biol 4: 842-854.

2. Li JJ, Li SA (2006) Mitotic kinases: the key to duplication, segregation, and cytokinesis errors, chromosomal instability, and oncogenesis. Pharmacol Ther 111: 974-984.

3. Kitzen JJ, de Jonge MJ, Verweij J (2010) Aurora kinase inhibitors. Crit Rev Oncol Hematol 73: 99-110.

4. Morozova O, Vojvodic M, Grinshtein N, Hansford LM, Blakely KM, et al. (2010) System-level analysis of neuroblastoma tumor-initiating cells implicates AURKB as a novel drug target for neuroblastoma. Clin Cancer Res 16: 4572-4582.

5. Fu J, Jiang, Q. & Zhang, C (2010) Collaboration of Mitotic Kinases in Cell Cycle Control. Nature Education 3(9):82.

6. Malumbres M, Barbacid M (2007) Cell cycle kinases in cancer. Curr Opin Genet Dev 17: 60-65.

7. Shapiro GI (2006) Cyclin-dependent kinase pathways as targets for cancer treatment. J Clin Oncol 24: 1770-1783.

8. Bettayeb K, Baunbaek D, Delehouze C, Loaec N, Hole AJ, et al. (2010) CDK Inhibitors Roscovitine and CR8 Trigger Mcl-1 Down-Regulation and Apoptotic Cell Death in Neuroblastoma Cells. Genes Cancer 1: 369-380.

9. Doble BW, Woodgett JR (2003) GSK-3: tricks of the trade for a multi-tasking kinase. J Cell Sci 116: 1175-1186.

10. Korur S, Huber RM, Sivasankaran B, Petrich M, Morin P, Jr., et al. (2009) GSK3beta regulates differentiation and growth arrest in glioblastoma. PLoS One 4: e7443.

11. Wakefield JG, Stephens DJ, Tavare JM (2003) A role for glycogen synthase kinase-3 in mitotic spindle dynamics and chromosome alignment. J Cell Sci 116: 637-646.

12. Wang Z, Smith KS, Murphy M, Piloto O, Somervaille TC, et al. (2008) Glycogen synthase kinase 3 in MLL leukaemia maintenance and targeted therapy. Nature 455: 1205-1209.

13. Dickey A, Schleicher S, Leahy K, Hu R, Hallahan D, et al. (2011) GSK-3beta inhibition promotes cell death, apoptosis, and in vivo tumor growth delay in neuroblastoma Neuro-2A cell line. J Neurooncol 104: 145-153.

14. Kotliarova S, Pastorino S, Kovell LC, Kotliarov Y, Song H, et al. (2008) Glycogen synthase kinase-3 inhibition induces glioma cell death through c-MYC, nuclear factor-kappaB, and glucose regulation. Cancer Res 68: 6643-6651.

15. Takagi M, Sueishi M, Saiwaki T, Kametaka A, Yoneda Y (2001) A novel nucleolar protein, NIFK, interacts with the forkhead associated domain of Ki-67 antigen in mitosis. J Biol Chem 276: 25386-25391.

16. Gruenbaum Y, Margalit A, Goldman RD, Shumaker DK, Wilson KL (2005) The nuclear lamina comes of age. Nat Rev Mol Cell Biol 6: 21-31.

17. Tsai MY, Wang S, Heidinger JM, Shumaker DK, Adam SA, et al. (2006) A mitotic lamin B matrix induced by RanGTP required for spindle assembly. Science 311: 1887-1893.

18. Morimoto H, Ozaki A, Okamura H, Yoshida K, Amorim BR, et al. (2007) Differential expression of protein phosphatase type 1 isotypes and nucleolin during cell cycle arrest. Cell Biochem Funct 25: 369-375.

19. Ginisty H, Sicard H, Roger B, Bouvet P (1999) Structure and functions of nucleolin. J Cell Sci 112 ( Pt 6): 761-772.

20. Srivastava M, Pollard HB (1999) Molecular dissection of nucleolin's role in growth and cell proliferation: new insights. FASEB J 13: 1911-1922.

21. Sif S, Stukenberg PT, Kirschner MW, Kingston RE (1998) Mitotic inactivation of a human SWI/SNF chromatin remodeling complex. Genes Dev 12: 2842-2851.

22. Andersen CL, Christensen LL, Thorsen K, Schepeler T, Sorensen FB, et al. (2009) Dysregulation of the transcription factors SOX4, CBFB and SMARCC1 correlates with outcome of colorectal cancer. Br J Cancer 100: 511-523.

23. Kohn KW (1999) Molecular interaction map of the mammalian cell cycle control and DNA repair systems. Mol Biol Cell 10: 2703-2734.
