# Supplementary figures and images for: Time-Lapse Imaging of Neuroblastoma Cells to Determine Cell Fate upon Gene Knockdown
Source: PLoS One. 2012 Dec 12;7(12):e50988. doi: 10.1371/journal.pone.0050988 (PMC3521006; doi:10.1371/journal.pone.0050988)

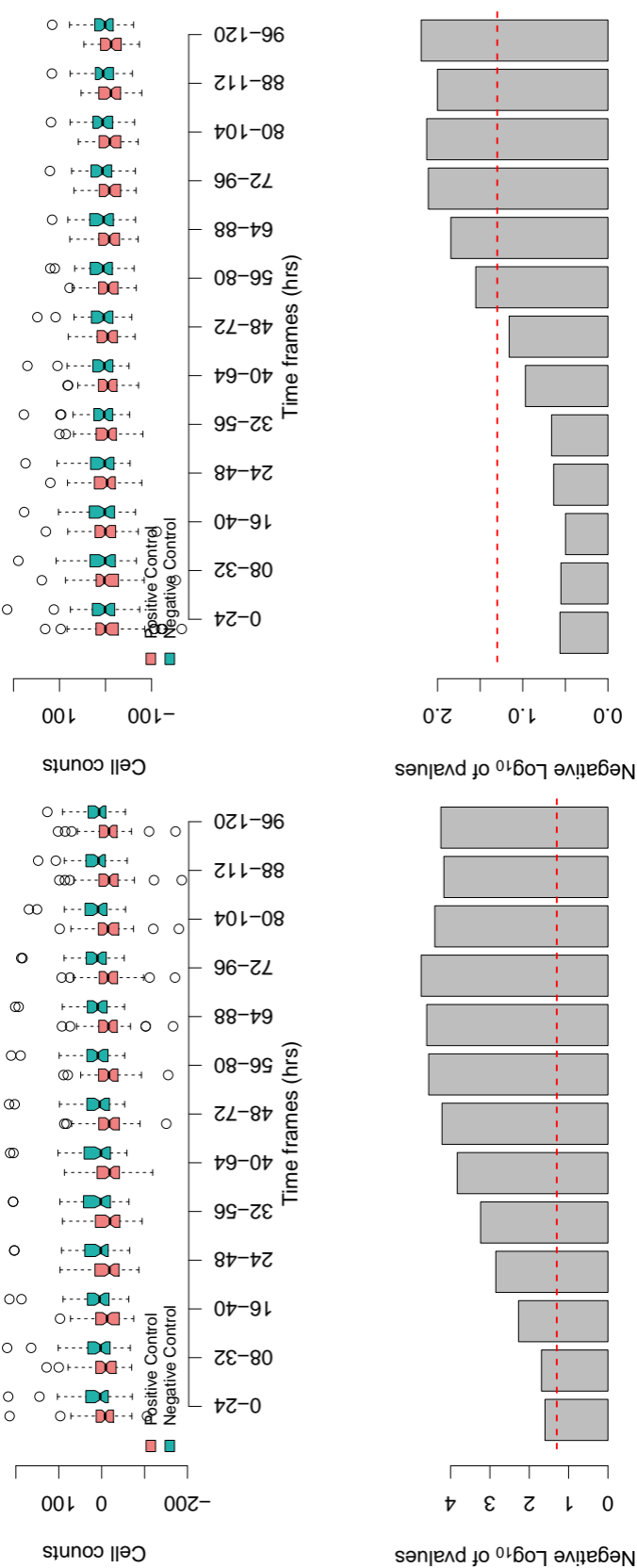

Supplement: Figure S1 — Experimental quality control. Top: cell counts of positive (coral red) and negative (coral blue) controls are plotted by boxplots for all time-frames. Bottom: Significance values of the differences of positive and negative controls are given for each time-frame by negative log10 p-values. A significance threshold (p-value = 0.05) is indicated by a red dashed line. (Left) SH-EP cell line: The positive controls show significant lower counts for all time-frames. (Right) SK-N-BE(2)-C cell line: The positive controls show significant lower counts for time frames ≥56 hrs. (PDF) [file pone.0050988.s002.pdf]

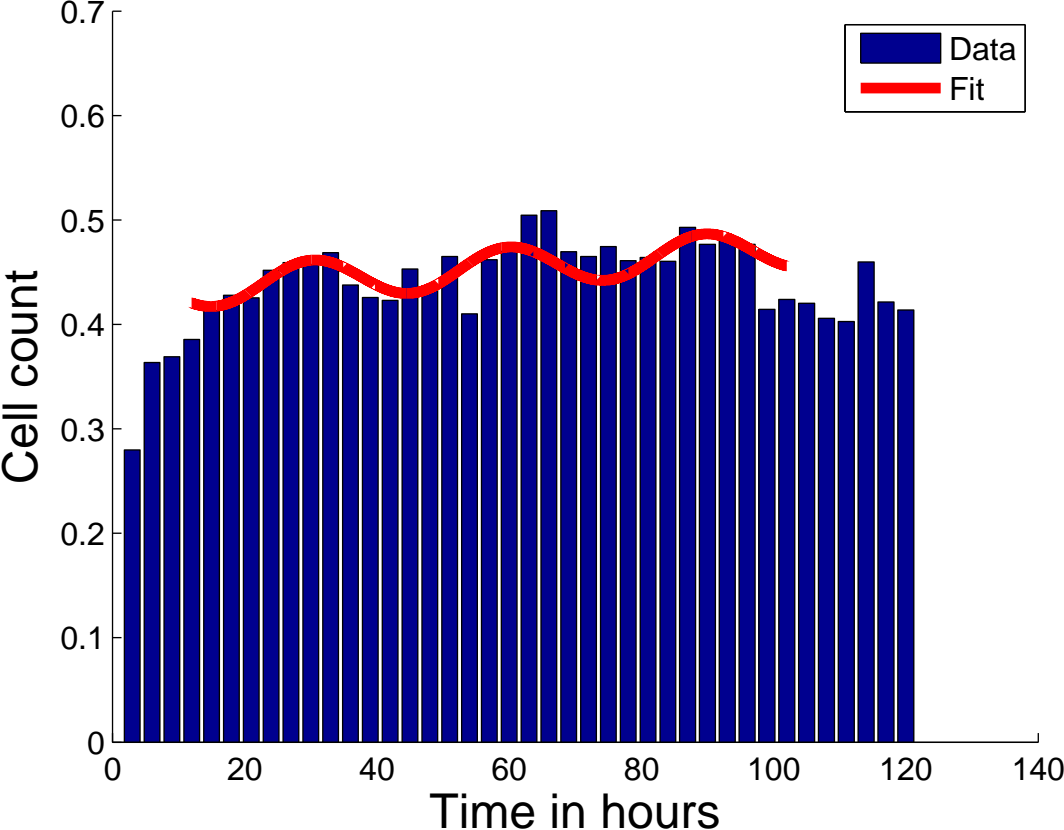

Supplement: Figure S2 — Time series of interphase cells during five days of screening of SK-N-BE(2)-C. The population showed a periodicity of ∼31 hours representing its cell cycle duration (blue bars: interphase counts of all screened cells for each time frame, red: fitting curve). (PDF) [file pone.0050988.s003.pdf]

## DLGAP5

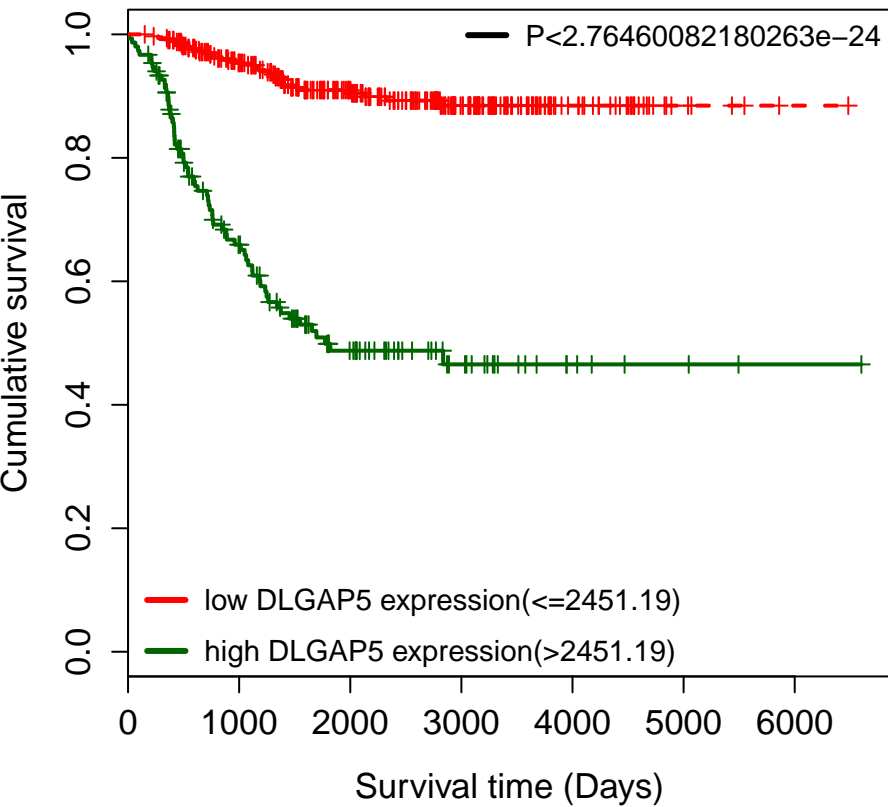

## SMO

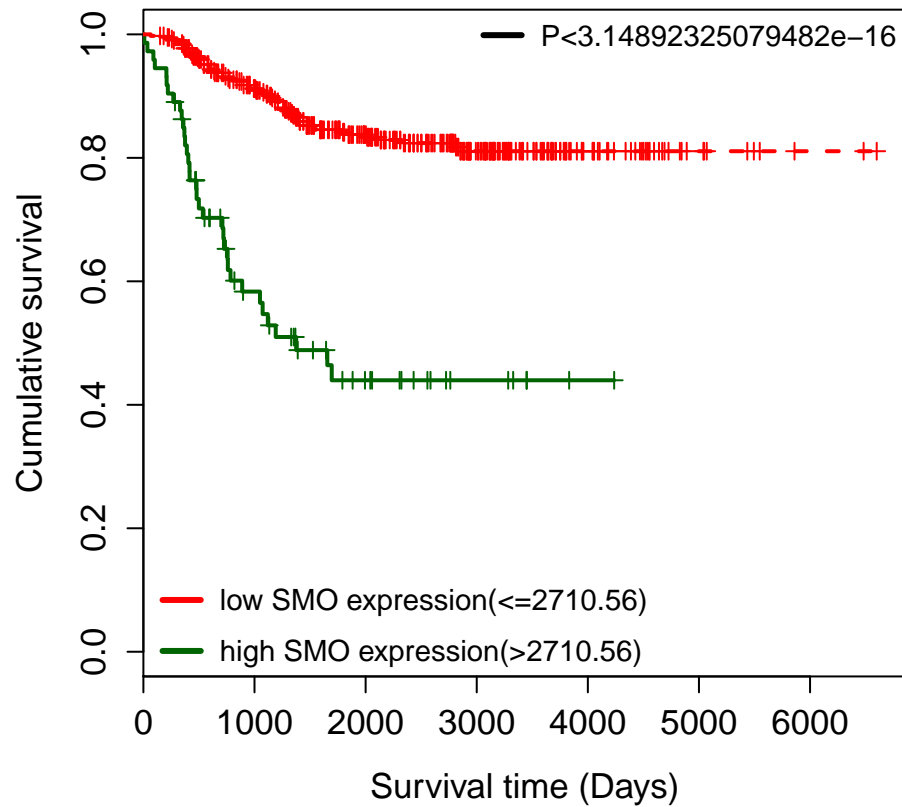

Supplement: Figure S3 — Kaplan Meier plots for two of the validated candidate genes (SMO and DLGAP5). The log-rank p-values are shown on the top right of the plots. (PDF) [file pone.0050988.s004.pdf]

DSCC1

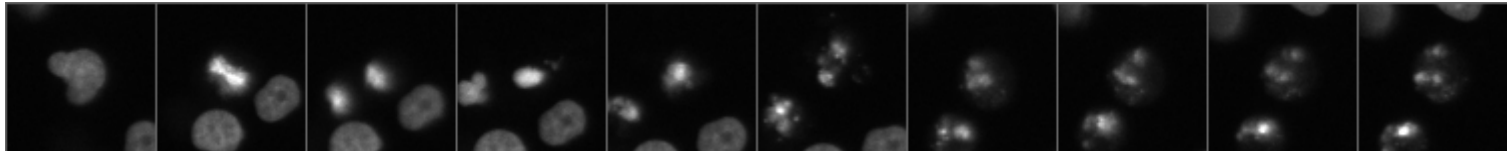

SSBP1

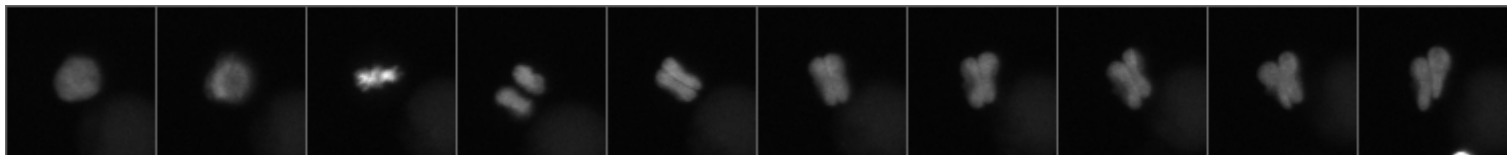

SNRPD1

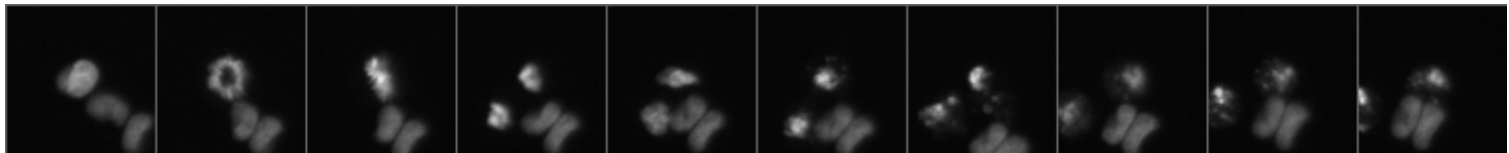

UBE2C

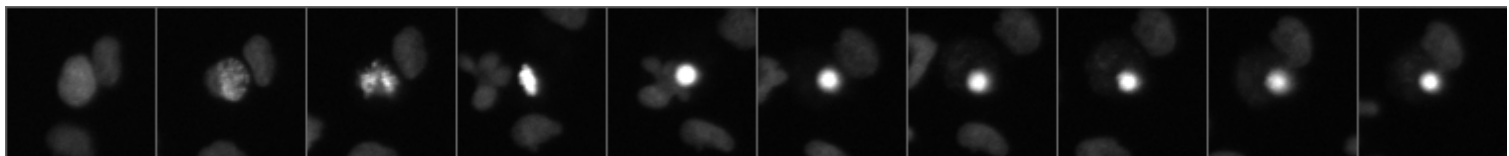

DLGAP5

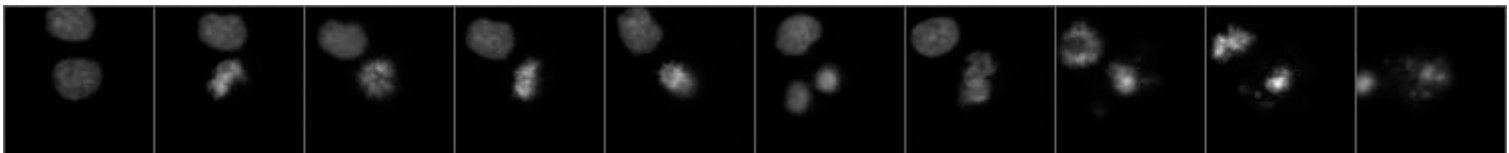

SMO

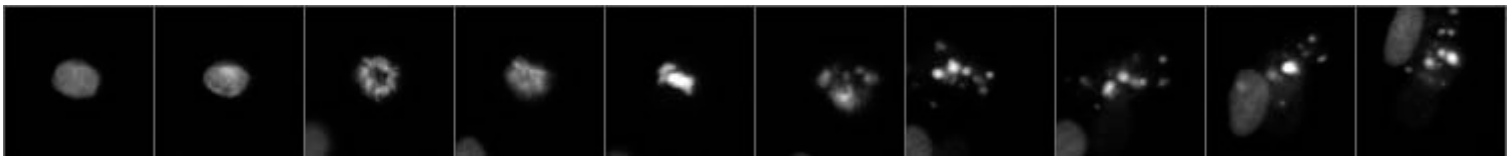

Supplement: Figure S4 — Selection of time-lapse images illustrating cell fate observed in the SH-EP cell line for the six validated genes. The image sequence of knockdown of DSCC1 shows a cell in interphase, mitosis (metaphase), interphase (daughter nuclei), deformation of the nucleus (cell death), and cell death. The sequence of knockdown of SSBP1 shows a cell in interphase, mitosis (prometaphase), mitosis (metaphase), mitosis (anaphase), and finally daughter nuclei sticking together in arrest. The sequence of knockdown of SNRPD1 shows a cell in interphase, mitosis (prometaphase), mitosis (metaphase), daughter nuclei and cell death. The sequence of knockdown of UBE2C shows a cell in interphase, mitosis (prometaphase), mitosis (anaphase), and cell death. The sequence of knockdown of DLGAP5 shows a cell in interphase, mitosis (metaphase), daughter nuclei, deformation, and cell death. The sequence of SMO knockdown shows a cell in interphase, mitosis (prometaphase), mitosis (metaphase) and cell death. (PDF) [file pone.0050988.s005.pdf]

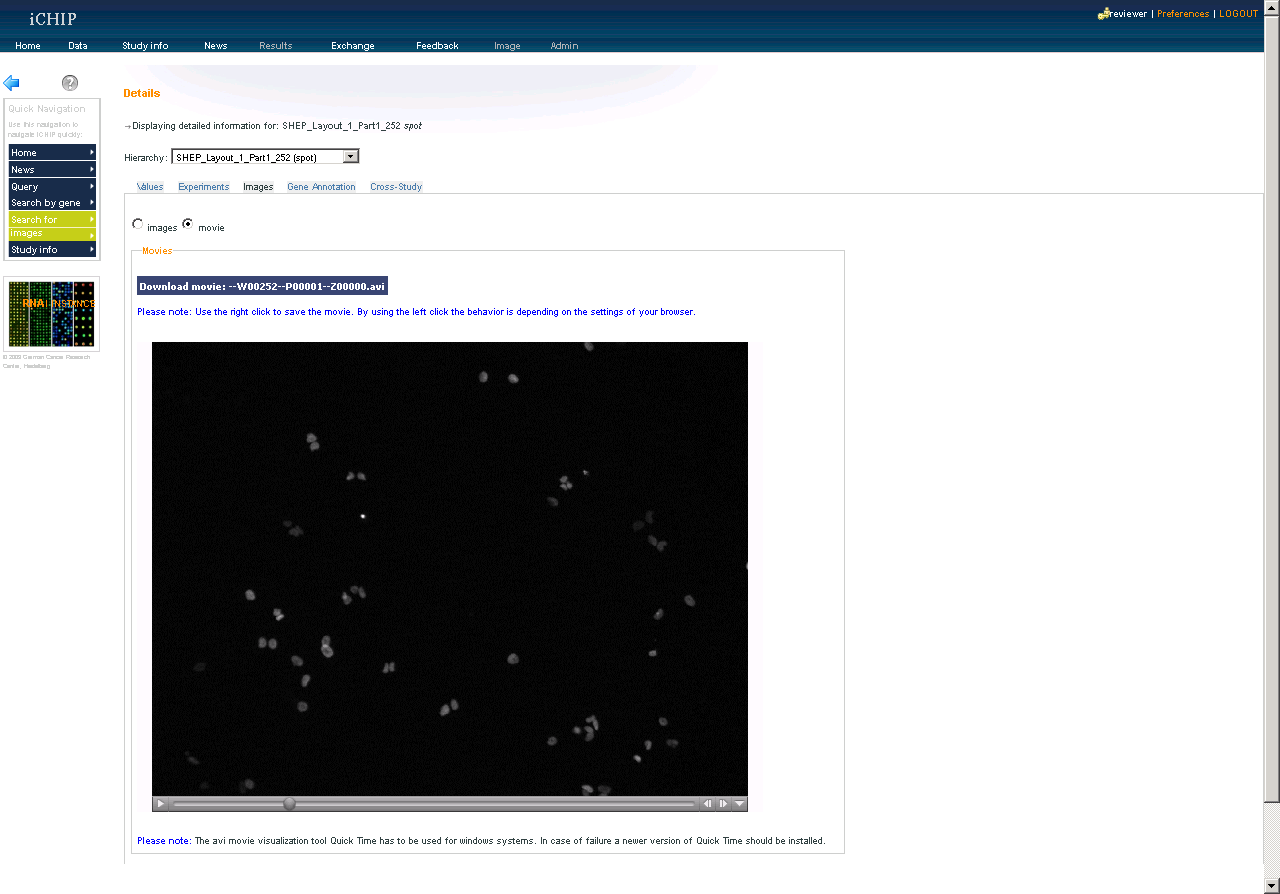

Supplement: Figure S5 — Screenshot of the web interface of the ichip database. Each movie and each image can be observed and downloaded. Access to the images is achieved by selection of a gene in the query page. Associated gene and siRNA information is also available as well as the calculated phenotpye scoring and related quality measures. (TIFF) [file pone.0050988.s006.tiff]

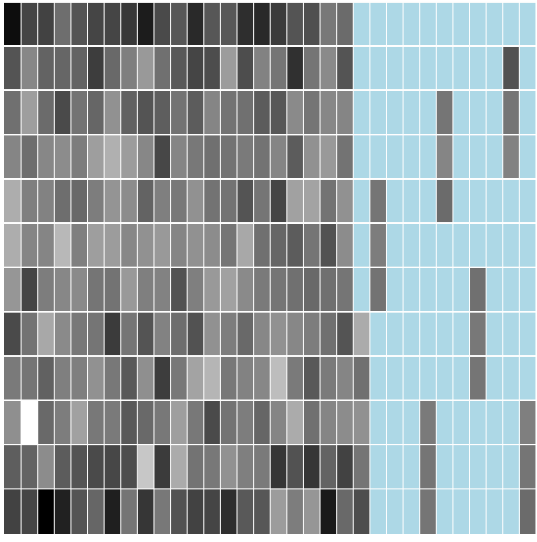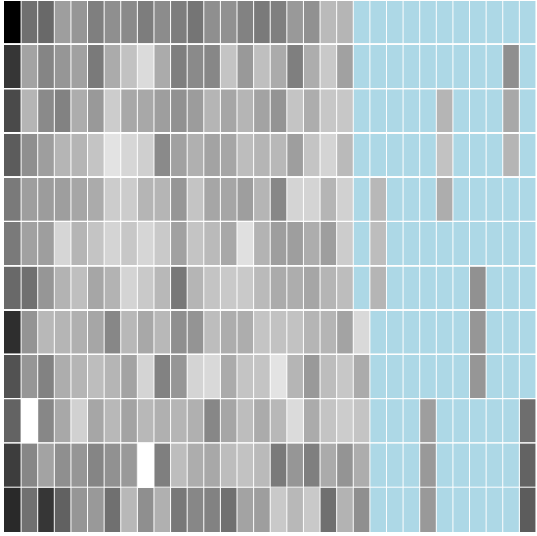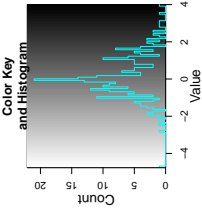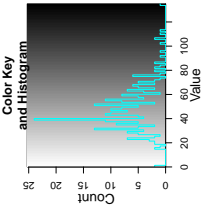

Supplement: Figure S6 — Cell arrays before and after normalization. The color key shows the distribution of the cell counts over the array. Left: A cell array before normalization, showing the edge effects with high cell counts in the most upper row. Right: The same cell array after B-score normalization. It shows a smoothing of the edge effects. Blue boxes represent empty spots which were not a part of the screen. (PDF) [file pone.0050988.s007.pdf]
