# Supplementary material for: Time-Lapse Imaging of Neuroblastoma Cells to Determine Cell Fate upon Gene Knockdown
Source: PLoS One. 2012 Dec 12;7(12):e50988. doi: 10.1371/journal.pone.0050988 (PMC3521006; doi:10.1371/journal.pone.0050988)
Supplement: Table S2 — Pathways of Reactome and gene groups from Gene Ontology which were enriched in the screened genes. (DOCX) [file pone.0050988.s009.docx]

**Supplementary Table S2.** Pathways of Reactome and gene groups from Gene Ontology which were enriched in the screened genes

| **ID** | **Term** | **Number of candidate genes in the pathway** | **P-value** |
| --- | --- | --- | --- |
| **Reactome Pathways** | | | |
| 152 | Cell cycle, mitotic | 37 | 4.26E-14 |
| 1538 | Cell cycle checkpoints | 14 | 4.78E-05 |
| 8017 | APC-CDC 20 mediated degradation of NEK2A | 5 | 0.0070 |
| 1698 | Metabolism of nucleotides | 8 | 0.030 |
| **Gene Ontology** | | | |
| 7067 | Mitosis | 47 | 7.61E-26 |
| 51301 | Cell division | 45 | 3.89E-22 |
| 7052 | Mitotic spindle organization | 09 | 5.66E-08 |
| 7093 | Mitotic cell cycle checkpoint | 11 | 1.15E-06 |
| 6260 | DNA replication | 23 | 1.49E-06 |
