# Supplementary material for: Time-Lapse Imaging of Neuroblastoma Cells to Determine Cell Fate upon Gene Knockdown
Source: PLoS One. 2012 Dec 12;7(12):e50988. doi: 10.1371/journal.pone.0050988 (PMC3521006; doi:10.1371/journal.pone.0050988)
Supplement: Table S3 — Confusion matrix for SH-EP cell line. (DOCX) [file pone.0050988.s010.docx]

**Supplementary Table S3a.** Confusion matrix for the training set (SH-EP)

|  | | **True class** | | | |
| --- | --- | --- | --- | --- | --- |
|  |  | **Interphase** | **Mitosis** | **Cell death** | **Artifact** |
|  | **Interphase** | 163 | 0 | 0 | 9 |
| **Predicted** | **Mitosis** | 3 | 79 | 21 | 0 |
| **class** | **Cell death** | 0 | 11 | 179 | 0 |
|  | **Artifact** | 4 | 0 | 0 | 106 |

**Supplementary Table S3b.** Confusion matrix for test set (set s) before automated correction (SH-EP)

|  | | **True class** | | | |
| --- | --- | --- | --- | --- | --- |
|  |  | **Interphase** | **Mitosis** | **Cell death** | **Artifact** |
|  | **Interphase** | 217 | 3 | 1 | 51 |
| **Predicted** | **Mitosis** | 59 | 27 | 27 | 40 |
| **class** | **Cell death** | 9 | 6 | 121 | 29 |
|  | **Artifact** | 26 | 0 | 6 | 117 |
|  | **Discarded** | 22 | 3 | 3 | 29 |
