# Supplementary material for: Time-Lapse Imaging of Neuroblastoma Cells to Determine Cell Fate upon Gene Knockdown
Source: PLoS One. 2012 Dec 12;7(12):e50988. doi: 10.1371/journal.pone.0050988 (PMC3521006; doi:10.1371/journal.pone.0050988)
Supplement: Table S4 — Confusion matrix for SK-N-BE(2)-C cell line. (DOCX) [file pone.0050988.s011.docx]

**Supplementary Table S4a.** Confusion matrix for the training set (SK-N-BE(2)-C)

|  | | **True class** | | | | |
| --- | --- | --- | --- | --- | --- | --- |
|  |  | **Interphase** | **Mitosis** | **Cell death** | **Artifact** | **Clusters^*^** |
|  | **Interphase** | 216 | 5 | 4 | 1 | 8 |
| **Predicted** | **Mitosis** | 5 | 57 | 25 | 0 | 0 |
| **class** | **Cell death** | 2 | 18 | 91 | 0 | 1 |
|  | **Artifact** | 1 | 0 | 0 | 43 | 0 |
|  | **Clusters** | 6 | 0 | 0 | 0 | 91 |

^*^For SK-N-BE(2)-C, artifact and cluster samples were separated into two groups (SH-EP: one common group)

**Supplementary Table S4b**. Confusion matrix for the test set (set s) before automated correction(SK-N-BE(2)-C)

|  | | **True class** | | | | |
| --- | --- | --- | --- | --- | --- | --- |
|  |  | **Interphase** | **Mitosis** | **Cell death** | **Artifact** | **Clusters** |
|  | **Interphase** | 127 | 11 | 24 | 24 | 68 |
|  | **Mitosis** | 1 | 3 | 1 | 2 | 3 |
| **Predicted** | **Cell death** | 55 | 9 | 83 | 48 | 97 |
| **class** | **Artifact** | 31 | 0 | 8 | 90 | 10 |
|  | **Clusters** | 14 | 2 | 11 | 4 | 10 |
|  | **Discarded** | 6 | 5 | 13 | 14 | 25 |

**Supplementary Table S4c.** Confusion matrix for test (set s) after automated correction (SK-N-BE(2)-C)

|  | | **True class** | | | | |
| --- | --- | --- | --- | --- | --- | --- |
|  |  | **Interphase** | **Mitosis** | **Cell death** | **Artifact** | **Clusters** |
|  | **Interphase** | 124 | 1 | 19 | 57 | 38 |
|  | **Mitosis** | 24 | 17 | 31 | 21 | 6 |
| **Predicted** | **Cell death** | 10 | 1 | 57 | 29 | 10 |
| **class** | **Artifact** | 29 | 7 | 21 | 88 | 9 |
|  | **Clusters** | 31 | 0 | 4 | 8 | 106 |
|  | **Discarded** | 16 | 4 | 8 | 10 | 13 |
