# Supplementary material for: Time-Lapse Imaging of Neuroblastoma Cells to Determine Cell Fate upon Gene Knockdown
Source: PLoS One. 2012 Dec 12;7(12):e50988. doi: 10.1371/journal.pone.0050988 (PMC3521006; doi:10.1371/journal.pone.0050988)
Supplement: Table S5 — Candidate genes with phenotype Cell death during or after mitosis. (DOCX) [file pone.0050988.s012.docx]

**Supplementary Table S5** Candidate genes with phenotype Cell death during or after mitosis.

| **SH-EP** | | **SK-N-BE(2)-C** | |
| --- | --- | --- | --- |
| **Gene Symbol** | **Entrez Id** | **Gene Symbol** | **Entrez Id** |
| AEN | 64782 | ANLN | 54443 |
| ATAD2 | 29028 | CDC2 | 983 |
| AURKA | 6790 | CYC1 | 1537 |
| C19orf48 | 84798 | CYCS | 54205 |
| CCNB1 | 891 | DCTPP1 | 79077 |
| CENPJ | 55835 | DDX21 | 9188 |
| CIT | 11113 | DLGAP5 | 9787 |
| CTSD | 1509 | DPH5 | 51611 |
| DLGAP5 | 9787 | DSCC1 | 79075 |
| DSCC1 | 79075 | ECSIT | 51295 |
| GNL3 | 26354 | EEF1E1 | 9521 |
| GOT1 | 2805 | ENO1 | 2023 |
| KARS | 3735 | ERCC6L | 54821 |
| LMNB1 | 4001 | FAM64A | 54478 |
| MKI67IP | 84365 | FASN | 2194 |
| MRPL3 | 11222 | GOT2 | 2806 |
| MTHFD2 | 10797 | HK2 | 3099 |
| NCL | 4691 | INCENP | 3619 |
| NEK2 | 4751 | KIF22 | 3835 |
| NT5DC2 | 64943 | MAD2L1 | 4085 |
| NUSAP1 | 51203 | MND1 | 84057 |
| RACGAP1 | 29127 | MRPS17 | 51373 |
| RFT1 | 91869 | NDC80 | 10403 |
| SMARCC1 | 6599 | NME2 | 4831 |
| SMO | 6608 | OIP5 | 11339 |
| SNRPD1 | 6632 | PLK1 | 5347 |
| SRM | 6723 | PNPT1 | 87178 |
| SSBP1 | 6742 | PPRC1 | 23082 |
| TP53 | 7157 | RAN | 5901 |
| UBE2C | 11065 | RRS1 | 23212 |
|  |  | SLC1A5 | 6510 |
|  |  | SMO | 6608 |
|  |  | SNRPD1 | 6632 |
|  |  | SSBP1 | 6742 |
|  |  | TOMM40 | 10452 |
|  |  | UBE2C | 11065 |
