# Supplementary material for: Time-Lapse Imaging of Neuroblastoma Cells to Determine Cell Fate upon Gene Knockdown
Source: PLoS One. 2012 Dec 12;7(12):e50988. doi: 10.1371/journal.pone.0050988 (PMC3521006; doi:10.1371/journal.pone.0050988)
Supplement: Table S6 — Results of the gene expression analysis for the six identified genes. (DOCX) [file pone.0050988.s013.docx]

**Supplementary Table S6** Results of the gene expression analysis for the six identified genes

| **Gene symbol** | **Up-regulation in state4-mycn-amplified in comparison to stage 1** | **Log rank test of the survival data** |
| --- | --- | --- |
| DSCC1 | 6.2e-18 | 3.7e-20 |
| DLGAP5 | 1.1e-17 | 5.4e-24 |
| UBEC2 | 3.5e-18 | 4.1e-26 |
| SSBP1 | 1.1e-15 | 7.3e-18 |
| SNPRD1 | 2.3e-21 | 3.4e-30 |
| SMO | 2.2e-15 | 7.3e-18 |
