# Supplementary material for: Time-Lapse Imaging of Neuroblastoma Cells to Determine Cell Fate upon Gene Knockdown
Source: PLoS One. 2012 Dec 12;7(12):e50988. doi: 10.1371/journal.pone.0050988 (PMC3521006; doi:10.1371/journal.pone.0050988)
Supplement: Table S7 — Kinase families and their predicted substrates from our candidate genes. (DOCX) [file pone.0050988.s014.docx]

**Supplementary Table S7.** Kinase families and their predicted substrates from our candidate genes

| **Kinase family** | **P-Value** | **Identified genes** |
| --- | --- | --- |
| **SH-EP** | | |
| AUR | 3.00E-04 | AURKA, TP53, RACGAP1 |
| GSK | 0.005 | MKI67IP, TP53, LMNB1, NCL, SMARCC1 |
| CDK | 0.006 | CCNB1, LMNB1, MYB, NCL, RACGAP1, SMARCC1, TP53 |
| RCK | 0.015 | SMARCC1, TP53, LMNB1, CIT |
| RSK | 0.013 | CCNB1, TP53 |
| PKA | 0.03 | TP53, LMNB1, AURKA |
| CAMLK | 0.044 | TP53, LMNB1 |
| MAPK | 0.044 | NEK2, TP53, LMNB1, NCL, SMARCC1 |
| **SK-N-BE(2)-C** | | |
| WEE | 0.0045 | CDC2, PLK1 |
| NEK | 0.021 | NDC80, RAN |
| AUR | 0.022 | NDC80, INCENP |
